# Inonotosis in Patient with Hematologic Malignancy

**DOI:** 10.3201/eid2401.171265

**Published:** 2018-01

**Authors:** Ana Fernández-Cruz, Mi Kwon, Jesús Guinea, Pilar Escribano, María del Carmen Martínez Jiménez, Ana Pulido, Verónica Parra, David Serrano, Jorge Gayoso, José Luis Díez Martín, Emilio Bouza

**Affiliations:** Hospital General Universitario Gregorio Marañón, Madrid, Spain (A. Fernández-Cruz, M. Kwon, J. Guinea, P. Escribano, M.C. Martínez Jiménez, A. Pulido, V. Parra, D. Serrano, J. Gayoso, J.L. Díez Martin, E. Bouza);; Instituto de Investigación Sanitaria Gregorio Marañón, Madrid (A. Fernández-Cruz, M. Kwon, J. Guinea, P. Escribano, M.D.C.M. Jiménez, A. Pulido, V. Parra, D. Serrano, J. Gayoso, J.L.D. Martin, E. Bouza);; CIBER de Enfermedades Respiratorias (CIBERES CB06/06/0058), Madrid (J. Guinea, E. Bouza)

**Keywords:** *Inonotus* spp., *Phellinus* spp., invasive fungal disease, hematological, febrile neutropenia, fungi, Spain

## Abstract

We report a lung-invasive fungal disease with possible cutaneous needle tract seeding in a patient with a febrile neutropenia caused by the Basidiomycetes mold *Inonotus* spp. Although rare, *Inonotus* spp. should be added to the list of microorganisms causing invasive fungal disease in neutropenic patients with hematologic malignancies.

A 33-year-old man in Madrid, Spain, with chronic myeloid leukemia in lymphoid blastic phase underwent allogeneic stem cell transplantation (SCT) from a matched unrelated donor in 2011. Four years later, he had an extramedullary pulmonary relapse, after which he began intensive reinduction chemotherapy. After the second cycle, prolonged severe aplasia developed in the patient. Invasive fungal disease (IFD) was suspected because of the presence of persistent fever despite broad-spectrum antimicrobial drugs and the appearance of a new pulmonary nodule (Figure 1; Technical Appendix) while the patient was receiving prophylactic micafungin (50 mg/d). Serologic fungal biomarkers were negative. A percutaneous pulmonary biopsy sample was taken, and empirical liposomal amphotericin B (3 mg/kg/d) was started (December 2015). Histology showed unspecific inflammatory tissue, and microbiology cultures were negative.

**Figure 1:**
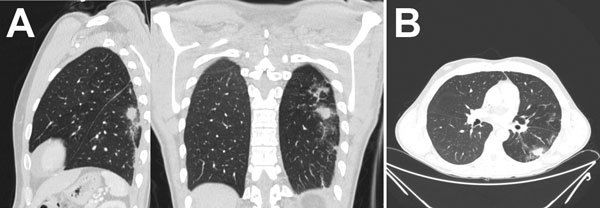
Computed tomography of the lungs in patient with invasive fungal disease caused by *Inonotus* spp., Madrid, Spain. Images show pulmonary nodule with halo sign in the left superior lobe, with peripheral distribution.

Salvage human leukocyte antigen–haploidentical SCT was performed in January 2016. Fever persisted during conditioning therapy, and a solitary cutaneous millimetric erythematous lesion appeared at the biopsy puncture site (Figure 2). Histopathology of the skin lesion showed dermal infiltration by periodic acid Schiff–positive elements compatible with fungal hyaline hyphae with parallel walls, regular septa, and branched hyphae with occasional bulb-like expansions; angioinvasion; and necrosis. Fungal culture of the specimen was negative, but panfungal PCR and further sequencing (*1*) revealed the presence of *Inonotus* spp. Voriconazole was added, and the lesion resolved in days. Neutrophil engraftment was achieved on day 12 post-SCT, with complete donor chimerism.

**Figure 2:**
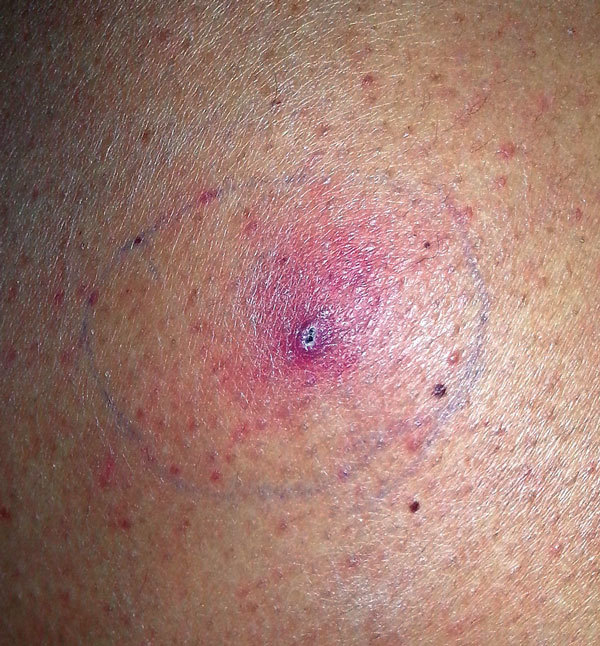
Skin lesion at biopsy site 4 weeks after the biopsy in patient with invasive fungal disease caused by *Inonotus* spp., Madrid, Spain.

On day 34, the pulmonary lesion progressed, but we could not prove IFD as the cause of concomitant pleural effusion. Despite intensified antifungal therapy, surgical debridement was required to resolve the empyema. The patient was discharged on oral posaconazole (300 mg/d) that was eventually replaced by micafungin.

A computed tomography scan performed 6 months after the SCT showed persistence of a single mass on the left lung inferior lobe together with a new hepatic nodule. We performed pulmonary segmentectomy, and lung histology showed mycetoma with fungal elements similar to those observed in the previous skin biopsy. The fungal culture yielded a fluffy, white, slow-growing mold. The lack of sporulation did not permit morphologic identification, although panfungal PCR and further sequencing again revealed the presence of *Inonotus* spp. in the lung tissue sample.

Filamentous Basidiomycetes molds are ubiquitous and able to colonize in patients with chronic pulmonary disease. They cause syndromes comparable to allergic bronchopulmonary or rhinosinusal aspergillosis. However, IFD caused by Basidiomycetes molds are extremely rare (*2*). It has been hypothesized that IFD could occur in patients with mycetoma if they become immunocompromised. Clinical presentation resembles that of other IFDs, with predominantly pulmonary involvement (Technical Appendix Table 1). Most filamentous Basidiomycetes are susceptible to antifungal drugs except for fluconazole and echinocandins. However, because many isolates do not sporulate, morphologic identification in the clinical microbiology laboratory is difficult without molecular techniques, and antifungal susceptibility testing is impossible to perform (*3*).

Invasive disease caused by *Inonotus* spp. (*Phellinus tropicalis* and *P. undulatus*) in humans has been described in 1 patient with diabetic nephropathy (*3*) and 6 patients with chronic granulomatous disease (*4*–*10*). Of note, 4 were breakthrough infections in patients receiving prophylactic itraconazole or posaconazole. Local infections had a favorable outcome; however, 1 patient with more extensive involvement had multiple relapses.

The infection in the patient we report mimicked other invasive mold infections in neutropenic patients with hematologic malignancies. However, we observed fungal invasion in the skin after the percutaneous puncture for the pulmonary biopsy, which suggests fungal seeding from the lung source during sample collection.

The presence of fungal elements invading the tissues supported the diagnosis of IFD; nevertheless, we did not initially consider *Inonotus* spp. to be the causative agent of the IFD in this patient because it rarely causes disease in humans. The clinical significance of the isolation of saprophytic molds in nonsterile clinical samples is difficult to ascertain. However, detection of *Inonotus* spp. in the lung tissue sample taken months after the skin lesion biopsy led us to reassess its potential role as an etiologic agent. In addition, the patient could have acquired the lung infection after inhalation of spores, and selective pressure of previous antimicrobial drugs could have triggered the breakthrough invasive *Inonotus* spp. infection. Antifungal therapy was selected without specific recommendations and without antifungal susceptibility testing (because of the poor sporulation of the isolate). Immunosuppression was more profound and prolonged than in other cases of IFD caused by *Inonotus* spp. Both surgery and antifungal therapy were required, and immunologic recovery, along with a subacute course, were probably essential for the favorable outcome of this patient.

In conclusion, *Inonotus* spp. should be added to the list of potential causal agents of IFD in neutropenic hematological patients. Systematic use of panfungal PCR targeting the internal transcribed tracer regions coupled with sequencing in patients at a high risk for IFD may be helpful for diagnosing rare invasive fungal infections.

**Technical Appendix.** Additional information about invasive fungal infections in neutropenic hematological patients.
