# Supplementary material for: Inonotosis in Patient with Hematologic Malignancy
Source: Emerg Infect Dis. 2018 Jan;24(1):180–2. doi: 10.3201/eid2401.171265 (PMC5749471; doi:10.3201/eid2401.171265)
Supplement: Supplementary file 1 — Technical Appendix. Additional information about invasive fungal infections in neutropenic hematological patients. [file 17-1265-Techapp-s1.pdf]

Article DOI: http://dx.doi.org/10.3201/eid2401.171265

Inonotosis in Patient with Hematologic Malignancy

Technical Appendix

Technical Appendix Table. Invasive fungal infections caused by Basidiomycetes in neutropenic hematological patients*

Author	Year	Place	Age, Sex	Species	Diagnostic technique	Underlying disease	Infection site	Therapy	Outcome	Comments
Nenoff P (*1*)	1997	Germany	40F	*Coprinus* spp *(Hormographiella aspergillata)*	Histology, culture	Relapsed ALL 5 y after HSCT	Lung	AmB	Fatal	Coinfection with *A. flavus* Breakthrough while on fluconazole
Verweij PE (*2*)	1997	Netherlands	24M	*Hormographiella aspergillata*	Histology, culture, PCR	ALL; HSCT; Relapse	Lung-multiple	AmB Itraconazole	Fatal	Postmortem diagnosis
Surmont I (*3*)	2002	Belgium	34F	*Hormographiella aspergillata*	Culture, PCR	NHL (lymphoblastic)	Lung abscess	AmB	Favorable	
Buzina W (*4*)	2005	Austria	9F	*Irpex lacteus*	Histology, culture, PCR	ALL	Lung abscesses	Voriconazole+AmB	Favorable	
Lagrou K (*5*)	2005	Belgium	34M	*Hormographiella aspergillata*	Histology, culture, PCR	AML; HSCT	Lung	Caspofungin	Fatal	Postmortem diagnosis.
Abuali M (*6*)	2009	NY (USA)	14F	*Hormographiella aspergillata*	Culture, PCR	AML; HSCT	Cutaneous (previous, palatal *Mucor*), probably disseminated (brain, lung cavitating lesions)	LAmB+caspofungin	Fatal	Mixed infection: first, palatal *Rhizomucor variabilis* Breakthrough while on Posaconazole and caspofungin
Conen A (*7*)	2010	Switzerland	41F	*Hormographiella aspergillata*	Histology, blood culture, PCR	AML; double HSCT	Lung nodules, eye, brain	Voriconazole Posaconazole Caspofungin	Fatal	GVHD Postmortem diagnosis.
			63F	*Hormographiella aspergillata*	Histology, culture, PCR	AML	Lung nodules	Voriconazole Posaconazole LAmB+surgery Voriconazole	Favorable, but death from underlying disease	
			55M	*Hormographiella aspergillata*	Histology, culture, PCR	AML; HSCT (double cord)	Lung nodules	LAmB+wedge resection Voriconazole	Fatal	Breakthrough while on voriconazole (previous proven aspergillosis).
Salit RB (*8*)	2010	Bethesda, MD, USA	41F	*Volvariella volvacea*	Histology, culture, PCR	HL; HSCT (cord)	Lung wedge infiltrates and CNS focal lesions	Voriconazole+LAmB	Fatal	

Author	Year	Place	Age, Sex	Species	Diagnostic technique	Underlying disease	Infection site	Therapy	Outcome	Comments
Pang KA (*9*)	2011	Strasbourg	25F	*Hormographiella aspergillata*	Histology, culture	ALL	Lung	Voriconazole, LAmB	Favorable	Breakthrough while on caspofungin)
Suárez F (*10*)	2011	Paris	23F	*Hormographiella aspergillata*	Histology, culture, PCR	Acute biphenotypic leukemia	Lung	Voriconazole LAmB	Favorable	Breakthrough while on caspofungin
			27M	*Hormographiella aspergillata*	Histology, culture, PCR	Adrenoleuko Dystrophy; HSCT (cord)	Lung, spleen	Caspofungin LAmB	Fatal	Postmortem diagnosis.
Bojic M (*11*)	2013	Austria	61F	*Hormographiella aspergillata*	Histology, culture, PCR	AML; AlloHSCT	Skin, lung nodules	Surgical debridement skin LAmB	Fatal	
Toya T (*12*)	2013	Japan	23F	*Schizophyllum commune*	Culture, PCR	ALL, SCT (cord)	Maxillary and etmoidal sinusitis	LAmB Voriconazole LAmB	Favorable	Breakthrough while on Itraconazole followed by micafungin
Heiblig M (*13*)	2015	France	19M	*Hormographiella aspergillata*	Histology, culture, PCR	Mitochondrial cytopathy MDS-AML; HSCT	Sino-orbito-cerebral involvement	Surgical debridement+ LAmB+Voriconazol e	Reduction of involvement, death from bacterial septic shock	Breakthrough while on Posaconazole (previous empirical caspofungin)
Corzo-León DE (*14*)	2015	Mexico	52F	*Hormographiella aspergillata*	Culture, PCR	AML; AlloHSCT	Lung	Voriconazole LAmB	Favorable, but died	Improvement, but died of unspecified cause
Nanno S (*15*)	2016	Japan	51M	*Hormographiella aspergillata*	Histology, culture, PCR	SMD-EB; Cord HSCT	Small intestine, lung and brain	LAmB+caspo	Fatal	Postmortem diagnosis, although previously detected in transbronchial biopsy, but not identified Breakthrough while on itraconazole
Lim DS (*16*)	2017	Singapore	1 M	*Earliella scabrosa*	Histology, culture, PCR	Aplastic anemia	Cutaneous emboli (multiple purpuric skin lesions), disseminated (pulmonary nodules	Not specified	Fatal	
Godet C (*17*)	2017	France	36M	*Hormographiella aspergillata*	Histology, PCR	AML. Cord HSCT	Lung nodule	Voriconazole Surgical resection LAmB+ Nebulized LAmB	Favorable	
Fernández-Cruz A (this study)	2017	Spain	33M	*Inonotus* spp	Histology, culture, PCR	ALL.	Lung nodule, skin	LAmB+vori Surgery Micafungin	Favorable	Breakthrough while on micafungin

*ALL, acute lymphoblastic leukemia; AlloHSCT, allogenic hematopietic stem cell transplantation; AmB, amphotericin B deoxicolate; AML, acute myeloblastic leukemia; HL, Hodgkin lymphoma; HSTC, hematopoietic stem cell transplant; LAmB, liposomal amphotericin B; MDS, myelodysplastic syndrome.

References

1. Nenoff P, Friedrich T, Schwenke H, Mierzwa M, Horn LC, Haustein UF. Rare fatal simultaneous mould infection of the lung caused by *Aspergillus flavus* and the basidiomycete *Coprinus* sp. in a leukemic patient. J Med Vet Mycol. 1997;35:65–9. PubMed http://dx.doi.org/10.1080/02681219780000901

2. Verweij PE, van Kasteren M, van de Nes J, de Hoog GS, de Pauw BE, Meis JF. Fatal pulmonary infection caused by the basidiomycete *Hormographiella aspergillata*. J Clin Microbiol. 1997;35:2675–8. PubMed

3. Surmont I, Van Aelst F, Verbanck J, De Hoog GS. A pulmonary infection caused by *Coprinus cinereus (Hormographiella aspergillata)* diagnosed after a neutropenic episode. Med Mycol. 2002;40:217–9. PubMed http://dx.doi.org/10.1080/mmy.40.2.217.219

4. Buzina W, Lass-Flörl C, Kropshofer G, Freund MC, Marth E. The polypore mushroom *Irpex lacteus*, a new causative agent of fungal infections. J Clin Microbiol. 2005;43:2009–11. PubMed http://dx.doi.org/10.1128/JCM.43.4.2009-2011.2005

5. Lagrou K, Massonet C, Theunissen K, Meersseman W, Lontie M, Verbeken E, et al. Fatal pulmonary infection in a leukaemic patient caused by *Hormographiella aspergillata*. J Med Microbiol. 2005;54:685–8. PubMed http://dx.doi.org/10.1099/jmm.0.46016-0

6. Abuali MM, Posada R, Del Toro G, Roman E, Ramani R, Chaturvedi S, et al. *Rhizomucor variabilis* var. regularior and *Hormographiella aspergillata* infections in a leukemic bone marrow transplant recipient with refractory neutropenia. J Clin Microbiol. 2009;47:4176–9. PubMed http://dx.doi.org/10.1128/JCM.00305-09

7. Conen A, Weisser M, Hohler D, Frei R, Stern M. *Hormographiella aspergillata*: an emerging mould in acute leukaemia patients? Clin Microbiol Infect. 2011;17:273–7. PubMed http://dx.doi.org/10.1111/j.1469-0691.2010.03266.x

8. Salit RB, Shea YR, Gea-Banacloche J, Fahle GA, Abu-Asab M, Sugui JA, et al. Death by edible mushroom: first report of *Volvariella volvacea* as an etiologic agent of invasive disease in a patient following double umbilical cord blood transplantation. J Clin Microbiol. 2010;48:4329–32. PubMed http://dx.doi.org/10.1128/JCM.01222-10

9. Pang KA, Godet C, Fekkar A, Scholler J, Nivoix Y, Letscher-Bru V, et al. Breakthrough invasive mould infections in patients treated with caspofungin. J Infect. 2012;64:424–9. PubMed http://dx.doi.org/10.1016/j.jinf.2011.12.015

10. Suarez F, Olivier G, Garcia-Hermoso D, Randriamalala E, Ghez D, Bruneau J, et al. Breakthrough *Hormographiella aspergillata* infections arising in neutropenic patients treated empirically with caspofungin. J Clin Microbiol. 2011;49:461–5. PubMed http://dx.doi.org/10.1128/JCM.01213-10

11. Bojic M, Willinger B, Rath T, Tobudic S, Thalhammer F, Böhm A, et al. Fatal skin and pulmonary infection caused by *Hormographiella aspergillata* in a leukaemic patient: case report and literature overview. Mycoses. 2013;56:687–9. PubMed http://dx.doi.org/10.1111/myc.12087

12. Toya T, Shinohara A, Tatsuno K, Seo S, Nannya Y, Ichikawa M, et al. A case of *Schizophyllum commune* sinusitis following unrelated cord blood transplantation for acute lymphoblastic leukemia. Int J Hematol. 2013;98:261–3. PubMed http://dx.doi.org/10.1007/s12185-013-1362-4

13. Heiblig M, Bozzoli V, Saison J, Thomas X, De Croze D, Traverse-Glehen A, et al.; Lyon HEMINF Study group. Combined medico-surgical strategy for invasive sino-orbito-cerebral breakthrough fungal infection with *Hormographiella aspergillata* in an acute leukaemia patient. Mycoses. 2015;58:308–12. PubMed http://dx.doi.org/10.1111/myc.12305

14. Corzo-León DE, Satlin MJ, Soave R, Shore TB, Schuetz AN, Jacobs SE, et al. Epidemiology and outcomes of invasive fungal infections in allogeneic haematopoietic stem cell transplant recipients in the era of antifungal prophylaxis: a single-centre study with focus on emerging pathogens. Mycoses. 2015;58:325–36. PubMed http://dx.doi.org/10.1111/myc.12318

15. Nanno S, Nakane T, Okamura H, Nishimoto M, Koh H, Nakamae H, et al. Disseminated *Hormographiella aspergillata* infection with involvement of the lung, brain, and small intestine following allogeneic hematopoietic stem cell transplantation: case report and literature review. Transpl Infect Dis. 2016;18:611–6. PubMed http://dx.doi.org/10.1111/tid.12561

16. Lim DS, Tan PL, Jureen R, Tan KB. Cutaneous emboli of invasive basidiomycosis in a child with aplastic anemia. Am J Dermatopathol. 2017;39:204–7. PubMed http://dx.doi.org/10.1097/DAD.0000000000000699

17. Godet C, Cateau E, Rammaert B, Grosset M, Le Moal G, Béraud G, et al. Nebulized liposomal amphotericin b for treatment of pulmonary infection caused by *Hormographiella aspergillata*: case report and literature review. Mycopathologia. 2017;182:709–13. PubMed http://dx.doi.org/10.1007/s11046-017-0117-9
